# Spinal cord microstructural changes are connected with the aberrant sensorimotor cortical oscillatory activity in adults with cerebral palsy

**DOI:** 10.1038/s41598-022-08741-9

**Published:** 2022-03-21

**Authors:** Michael P. Trevarrow, Anna Reelfs, Sarah E. Baker, Rashelle M. Hoffman, Tony W. Wilson, Max J. Kurz

**Affiliations:** 1 Institute for Human Neuroscience, Boys Town National Research Hospital, 14090 Mother Teresa Lane, Boys Town, NE 68010 USA; 2 Department of Physical Therapy, Creighton University, Omaha, NE USA

**Keywords:** Motor control, Spine regulation and structure

## Abstract

Previous animal models have illustrated that reduced cortical activity in the developing brain has cascading activity-dependent effects on the microstructural organization of the spinal cord. A limited number of studies have attempted to translate these findings to humans with cerebral palsy (CP). Essentially, the aberrations in sensorimotor cortical activity in those with CP could have an adverse effect on the spinal cord microstructure. To investigate this knowledge gap, we utilized magnetoencephalographic (MEG) brain imaging to quantify motor-related oscillatory activity in fourteen adults with CP and sixteen neurotypical (NT) controls. A subset of these participants also underwent cervical-thoracic spinal cord MRI. Our results showed that the strength of the peri-movement beta desynchronization and the post-movement beta rebound were each weaker in the adults with CP relative to the controls, and these weakened responses were associated with poorer task performance. Additionally, our results showed that the strength of the peri-movement beta response was associated with the total cross-sectional area of the spinal cord and the white matter cross-sectional area. Altogether these results suggest that the altered sensorimotor cortical activity seen in CP may result in activity-dependent plastic changes within the spinal cord microstructure, which could ultimately contribute to the sensorimotor deficits seen in this population.

## Introduction

Cerebral palsy (CP) refers to a group of movement disorders that are a result of an insult to the developing brain^1^. The disorder is primarily characterized by musculoskeletal impairments, although recent literature has identified that deficits of sensation and perception are also an integral component of the disorder^2–6^. While CP is not considered a neurologically progressive disorder, accelerated sarcopenia, weight gain, sedentary lifestyles, and balance deficits result in a progressive deterioration of the musculoskeletal system^7–14^. This progressive decline may result in increased difficulty in performing upper extremity tasks of daily living (brushing teeth, buttoning shirt)^8^. Although a wide breadth of occupational therapeutic approaches have been employed to improve the hand motor actions of individuals with CP, very few of these approaches are considered “green light” therapies^15^. The underlying issue stems at least partially from a lack of understanding of the basic neurophysiological components that contribute to the altered upper extremity motor actions of individuals with CP.

Previous neuroimaging studies have established that there are stage-dependent changes in the strength of sensorimotor cortical oscillations within the beta (15–30 Hz) and gamma bands (> 30 Hz) when planning and executing hand motor actions. Prior to movement onset, there is a power decrease in the beta band which is sustained throughout the movement duration^16–20^. This so-called peri-movement beta event-related desynchronization (ERD) has been linked to motor planning, begins earlier for easier movements, and its relative strength appears to reflect the certainty of the motor action to be completed^20–30^. After movement completion, there is an increase in the beta power, termed the post-movement beta rebound (PMBR)^16,18–20,22–25^, which is thought to reflect either afferent feedback to the motor cortices^31,32^, active inhibition of cortical networks after movement termination^33–35^, or confidence in the motor actions executed based on the internal model^36,37^. Finally, there is a transient gamma event-related synchronization (ERS) that is tightly yoked to movement onset^20,38–40^. Historically, the gamma ERS has been seen as critical for the initialization and execution of motor commands^18,38,41–43^. However, more recent studies have also linked changes in the strength of the gamma ERS with the certainty of motor responses and response selection in the context of interference^21,44–46^. Altogether, these seminal neurophysiological studies have highlighted that the strength and other properties of motor-related beta and gamma oscillations are directly related to cognitive-motor performance.

Prior clinical investigations have shown that the strength of beta and gamma sensorimotor cortical oscillations are altered in youth with CP. Specifically, several investigations have reported that the beta ERD within the primary motor cortex, the premotor cortex, and the supplementary motor area is stronger in youth with CP relative to age-matched controls during leg movements^47–49^. Furthermore, the gamma ERS has been shown to be weaker in youth with CP during a lower extremity motor task. Fewer studies have evaluated whether these altered cortical oscillations extend to hand motor actions in youth with CP. In fact, only one study to date has quantified the strength of the PMBR and gamma ERS during upper extremity movements, and this study indicated weaker responses in youth with CP during performance of an arrow-based version of the Eriksen flanker task^50^. Although these investigations have advanced our understanding of the impact of CP on sensorimotor cortical oscillations, we still have major knowledge gaps in our understanding of these cortical oscillations, especially in adults with CP, despite the evidence that there is a decline in hand motor function throughout adulthood^8^.

While an understanding of how the cortical activity contributes to the altered hand motor actions is imperative, recent fMRI results have demonstrated that the fidelity of the hand motor actions is also dependent upon coherence between the cortex and the spinal cord^51^. Thus, the spinal cord also likely plays a prominent role in the uncharacteristic hand motor actions seen in individuals with CP. Using an animal model, Friel and colleagues (2007) demonstrated that inactivation of one hemisphere of the brain early in development can result in structural changes within the spinal cord. Specifically, the non-affected hemisphere may assume control over the paretic limb, and the descending motor tract terminations shift towards occupying tissue in the dorsal horn of the spinal cord^52,53^. Thus, an insult to the developing brain can result in the motor tracts terminating in areas that are normally occupied by the sensory neurons. In turn, this may result in activity-dependent competition for space in the dorsal horn of the spinal cord and lead to overall pruning of the corticospinal and sensory neurons. Our recent structural imaging results have supported this premise by showing that the cross-sectional area (CSA) of the spinal cord and proportion of gray matter are markedly reduced in adults with CP, and that these structural changes predict reduced hand dexterity^54^. Based on the prior animal work of Friel et al. (2007), we suspect that these microstructural changes are at least partially linked with the altered cortical activity seen in individuals with CP. However, this conjecture has yet to be tested.

Herein, we sought to address these knowledge gaps by using a multimodal neuroimaging approach. We utilized the high temporal and spatial precision of MEG to investigate motor-related oscillations associated with performance of an arrow-based version of the Eriksen Flanker task. This cognitive-motor task was chosen as prior studies have demonstrated that it’s associated with robust beta and gamma oscillatory activity^44,50^. Secondarily, we utilized MRI to investigate how the spinal cord microstructure may be related to the strength of sensorimotor cortical activity. Based on the prior literature, we hypothesized that the beta ERD, PMBR, and gamma ERS would each be altered in adults with CP relative to their demographically-matched peers. In addition, we hypothesized that the fidelity of the spinal cord microstructure would be significantly related to the altered sensorimotor cortical oscillatory activity.

## Methods

### Participants

Adult participants with CP and demographically matched neurotypical (NT) controls were recruited to participate in this cohort investigation. Individuals with CP were excluded from participating in this investigation if they had an orthopedic surgery or anti-spasticity treatments within the last 6 months or clinical diagnosis of an arterial ischemic stroke or middle cerebral artery stroke. The respective stroke cases were not included because they are associated with large volume loss that would likely impact the cortical surface. Participants were also excluded according to MEG/MRI exclusionary criteria such as metal implants, dental braces or permanent retainers, or other metallic or otherwise magnetic non-removable devices. None of the participants had a prior history of epilepsy or seizure activity. Each participant provided written informed consent to participate in the investigation. The protocol for this investigation was approved by the Institutional Review Board and was in compliance with the Code of Ethics of the World Medical Association. Effectively, all the methods used in this investigation were performed in accordance with the relevant guidelines and regulations.

### Experimental paradigm

**Figure 1:**
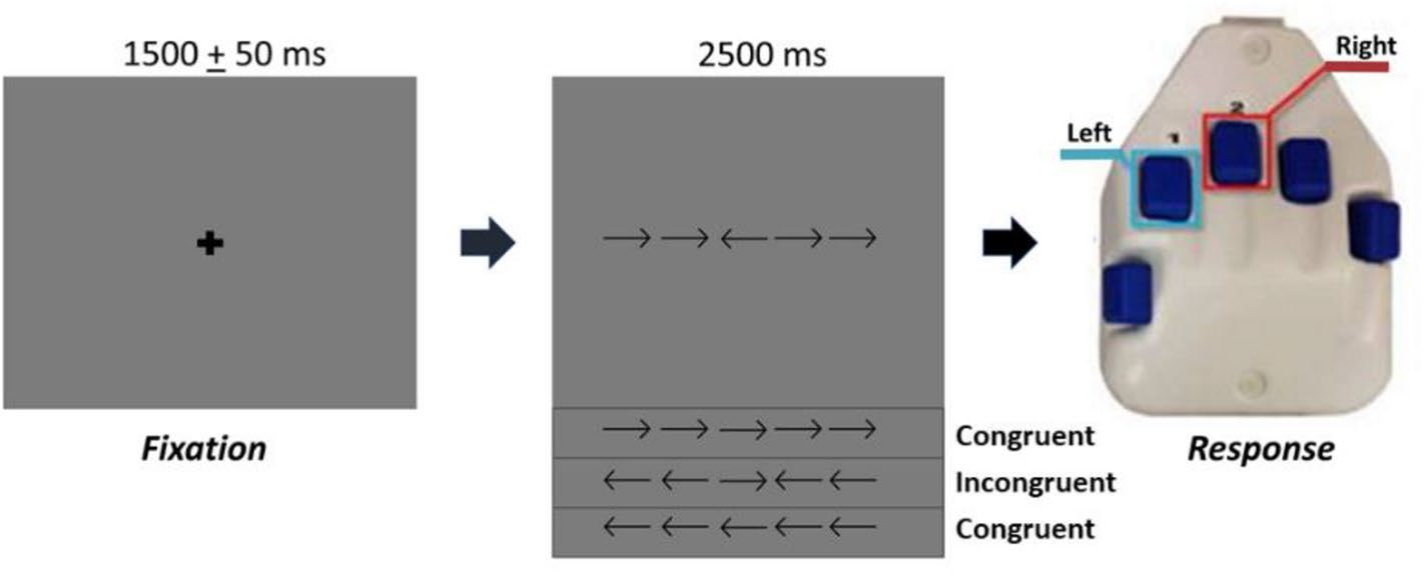
Flanker task design. For each trial, the participants fixated on a crosshair for 1500 ± 50 ms, then a series of five arrows appeared for 2500 ms. Upon arrows appearing, the participants responded with their right hand regarding the center arrow pointing to the left (2nd digit) or right (3rd digit). In the congruent condition, the flanking arrows pointed in the same direction as the middle arrow. In the incongruent condition, the flanking arrows pointed in the opposite direction than the middle arrow.

During MEG recording, participants were seated in a nonmagnetic chair with their right hand positioned on a custom-made five-finger button pad. Each button press sent a unique signal (i.e., TTL pulse/trigger code) to the MEG acquisition computer, and thus behavioral responses were temporally synced with the MEG data. The participants completed an arrow-based version of the Eriksen flanker task^55,56^. Each trial began with a fixation cross that was presented for an interval of 1500 ± 50 ms. A row of five arrows was then presented for 2500 ms and the participants were instructed to respond about the direction of the middle target arrow with their second (left arrow) or third (right arrow) digit of the right hand using the custom 5-button pad (Fig. 1). Visual presentation consisted of either a series of flanking arrows that had directions that were congruent (i.e., same direction) or incongruent (i.e., opposite direction) of the middle target arrow. The task stimuli were visually projected onto a screen that was approximately one meter from the participant. A total of 200 trials were presented, making the overall MEG recording time about 14 min. Trials were equally split and pseudo-randomized between congruent and incongruent conditions, with left and right pointing arrows being equally represented in each condition. Only correct responses were included for further analysis.

### MEG acquisition parameters and coregistration with MRI

All recordings were conducted in a one-layer magnetically-shielded room with active shielding engaged. Neuromagnetic responses were sampled continuously at 1 kHz with an acquisition bandwidth of 0.1–330 Hz using an MEGIN/Elekta MEG system with 306 magnetic sensors (Helsinki, Finland). Using MaxFilter (v2.2; Elekta), MEG data from each subject were individually corrected for head motion and subjected to noise reduction using the signal space separation method with a temporal extension^57,58^.

Prior to starting the MEG experiment, four coils were attached to the subject’s head and localized, together with the three fiducial points and scalp surface, with a 3-D digitizer (Fastrak 3SF0002, Polhemus Navigator Sciences, Colchester, VT, USA). Once the subject was positioned for MEG recording, an electric current with a unique frequency label (e.g., 322 Hz) was fed to each of the coils. This induced a measurable magnetic field and allowed for each coil to be localized in reference to the sensors throughout the recording session. Since the coil locations were also known in head coordinates, all MEG measurements could be transformed into a common coordinate system. With this coordinate system, each participant’s MEG data were coregistered with structural T1-weighted MRI data prior to source space analyses using BESA MRI (Version 2.0). Structural T1-weighted MRI images were acquired using a Siemens Prisma 3-Tesla MRI scanner with a 64-channel head/neck coil and a sequence with the following parameters: TR = 2400 ms; TE = 1.96 ms; flip angle = 8°; FOV = 256 mm; slice thickness = 1 mm (no gap); voxel size = 1 × 1 × 1 mm. These data were aligned in parallel to the anterior and posterior commissures and transformed into standardized space. Each participant’s 4.0 × 4.0 × 4.0 mm MEG functional images were transformed into standardized space using the transform that was previously applied to the structural MRI volume and spatially resampled.

### MEG time–frequency transformation and statistics

Cardiac artifacts were removed from the data using signal-space projection (SSP), which was accounted for during source reconstruction^59^. The continuous magnetic time series was divided into epochs of 4000 ms duration from − 2000 ms to 2000 ms, with the baseline being defined as − 1600 to − 800 ms and 0.0 ms being movement onset (i.e., button press). Epochs containing artifacts (e.g., eye blinks, muscle artifacts, etc.) were rejected based on a fixed-threshold method using individual amplitude and gradient thresholds, supplemented with visual inspection. The number of trials used in the final analyses did not statistically differ by group or condition (P’s > 0.05).

Artifact-free epochs were transformed into the time–frequency domain using complex demodulation, and the resulting spectral power estimations per sensor were averaged over trials to generate time–frequency plots of mean spectral density. These sensor-level data were normalized using the respective bin’s baseline power, which was calculated as the mean power during the − 1600 to − 800 ms time period. The specific time–frequency windows used for imaging were determined by statistical analysis of the sensor-level spectrograms across the entire array of gradiometers. To reduce the risk of false positive results while maintaining reasonable sensitivity, a two-stage procedure was followed to control for Type 1 error. In the first stage, paired t-tests against baseline were conducted on each data point and the output spectrogram of t-values was thresholded at P < 0.05 to define time–frequency bins containing potentially significant oscillatory deviations relative to baseline across all participants. In stage two, time–frequency bins that survived the threshold were clustered with temporally and/or spectrally neighboring bins that were also above the P < 0.05 threshold, and a cluster value was derived by summing all of the t-values of all data points in the cluster. Nonparametric permutation testing was then used to derive a distribution of cluster-values, and the significance level of the observed clusters (from stage one) were tested directly using this distribution^60,61^. For each comparison, at least 1,000 permutations were computed to build a distribution of cluster values. Based on these analyses, the time–frequency windows that corresponded to events of a priori interest (i.e., the beta ERD, PMBR, and gamma ERS) and contained significant oscillatory events across all participants and conditions were subjected to the beamforming analysis.

### MEG imaging & statistics

Cortical networks were imaged using an extension of the dynamic imaging of coherent sources (DICS) beamformer^62,63^, which employs spatial filters in the time–frequency domain to calculate source power for the entire brain volume. The single images were derived from the cross-spectral densities of all combinations of MEG gradiometers averaged over the time–frequency range of interest, and the solution of the forward problem for each location on a grid specified by input voxel space. Following convention, we computed noise-normalized, source power per voxel in each participant using active (i.e., task) and passive (i.e., baseline) periods of equal duration and bandwidth^62,64^. Such images are typically referred to as pseudo-*t* maps, with units (pseudo-*t*) that reflect noise-normalized power differences (i.e., active vs. passive) per voxel. MEG pre-processing and imaging used the Brain Electrical Source Analysis (BESA version 6.1) software.

Normalized differential source power was computed for the statistically-selected time–frequency bands (see below) over the entire brain volume per participant at 4.0 × 4.0 × 4.0 mm resolution. The resulting 3D maps of brain activity were averaged across participants to assess the neuroanatomical basis of significant oscillatory responses identified through the sensor-level analysis. We then extracted virtual sensors (i.e., voxel time series) for the peak voxel of each oscillatory response. To compute the virtual sensors, we applied the sensor weighting matrix derived through the forward computation to the preprocessed signal vector, which yielded a time series corresponding to the location of interest. Note that this virtual sensor extraction was done per participant, once the coordinates of interest (i.e., one per cluster) were known. Once these virtual sensors were extracted, the magnitude of the beta ERD, PMBR, and gamma ERS were calculated as the minimum (for the beta ERD) and maximum (for the PMBR and gamma ERS) amplitude within the target period of interest.

### Motor behavioral data

The output of the button pad was simultaneously collected at 1 kHz along with the MEG data. All 200 trials were included for the analysis of the behavioral data. Accuracy was defined as the number of correct responses divided by the total number of trials. The time the participant took to decide the direction of the target arrow (i.e., reaction time) was calculated based on the time from when the arrow array was presented to when the button was pressed.

### Spinal cord MRI processing

A portion of this investigation follows up on an MRI spinal cord project that was published previously^54^. The partially automated Spinal Cord Toolbox (version 3.2.2, Polytechnique, Montreal, Canada)^65^ was used to process the spinal cord images, and the parameters of each sequence are fully described in this previous study. In brevity, cervical-thoracic spinal cord MRI scans were acquired with a Siemens Prisma 3 T scanner equipped with a 64-channel head/neck coil. After vertebral labeling, the spinal cord was initially straightened, as described by De Leener et al.^66^ which was then followed by inferior-superior affine alignment based on vertebral levels. The spinal cord centerline was then aligned between the template and the participant using the center of mass of the spinal cord segmentation, which was then followed by a non-linear within-plane BSplineSyN registration^67^. The spinal cord internal structure was accounted for by assuming a linear deformation based on the outer shape of the spinal cord. The calculated total CSA across C6—T3 was extracted from the T2 images and the T2* was used for gray and white matter extraction. Furthermore, the PAM50 template was registered to the diffusion-weighted images and magnetization transfer (MT) images after motion correction, and the diffusion tensors for the respective spinal cord tracts were calculated. As the task was performed with the right hand, the fractional anisotropy (FA) and magnetization transfer ratio (MTR) values from the right corticospinal tract (CST) and cuneatus tracts were subsequently calculated from the diffusion-weighted images. These respective values were used to evaluate the relationship between the strength of the cortical oscillations and the integrity of the spinal cord microstructure. Complete details of the spinal cord imaging acquisition parameters and processing pipelines are detailed in Trevarrow et al.^54^.

### Statistical analysis

We performed 2 × 2 × 2 mixed model ANOVAs with condition (congruent and incongruent) as a within subjects factor, group (CP and NT) as a between subjects factor, and sex (male and female) as a between subjects factor in order to determine group, condition, and sex differences and identify possible interactions with respect to reaction time and accuracy within the task. Sex was included as an exploratory variable in the model since CP has been reported to be more common in males than females, but presentation differences are less understood^68^. For the motor-related oscillations, we utilized 2 × 2 × 2 ANOVAs with condition (congruent and incongruent) as a within subjects factor, group (CP and NT) as a between subjects factor, and sex (male and female) as a between subjects factor in order to determine any group, condition, or sex main effects, as well as interactions with respect to motor-related oscillatory activity. Lastly, Pearson correlations were used to determine the relationship between the behavioral data and strength of the beta responses, as well as the spinal cord structural MRI measures and the MEG/behavioral measures. All statistical analyses were conducted at a 0.05 alpha level. Results are reported as mean ± standard error of the mean.

## Results

### Participants

The participants included in this cohort study consisted of fourteen adults with CP (Age = 33.1 ± 8.6 years, Range 20–47 yrs, MACS = I–IV, Females = 8) and sixteen NT controls (Age = 33.3 ± 9.8 yrs, Range 19–49 yrs, Females = 9). Further description of the participant demographics is shown in Table 1. The two groups did not statistically differ by age (P = 0.962). The participants with CP had Manual Ability Classification System (MACS) levels between I-IV. MACS level of I indicates that the participant can easily handle most objects, while higher MACS levels indicate increased difficulty in handling objects.

**Table 1:** Characteristics of participants with cerebral plasy.

Characteristics of participants	n = 14
Age, mean (SD)	33.1 (8.6)
Males, n (%)	6 (43%)
**Type of cerebral plasy, n (%)**	
Hemiplegia	3 (21%)
Diplegia	11 (79%)
**MACS, n (%)**	
Level I	6 (43%)
Level II	6 (43%)
Level II	1 (7%)
Level IV	1 (7%)

*MACS* Manual Ability Classification System.

### Motor behavioral results

**Figure 2:**
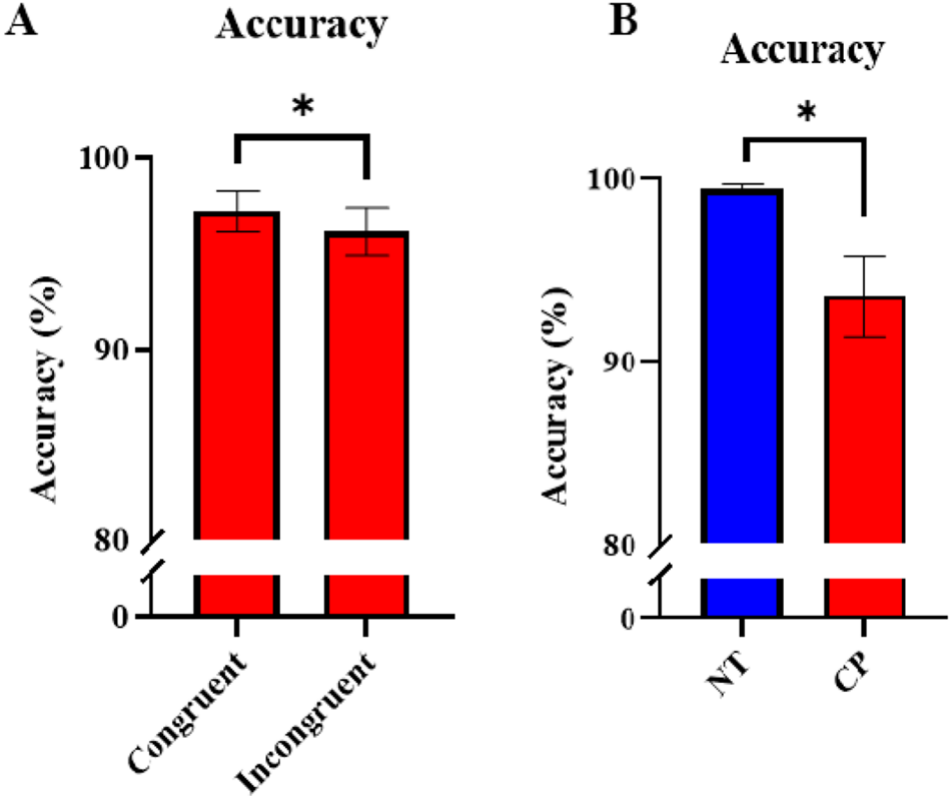
Accuracy between groups and conditions in the flanker task. (**A**) Accuracy (in %) was higher in the congruent versus the incongruent condition across all participants. (**B**) Accuracy was also higher in the neurotypical (NT) controls compared with the individuals with cerebral palsy (CP). Error bars reflect the standard error of the mean. *P < 0.05.

**Figure 3:**
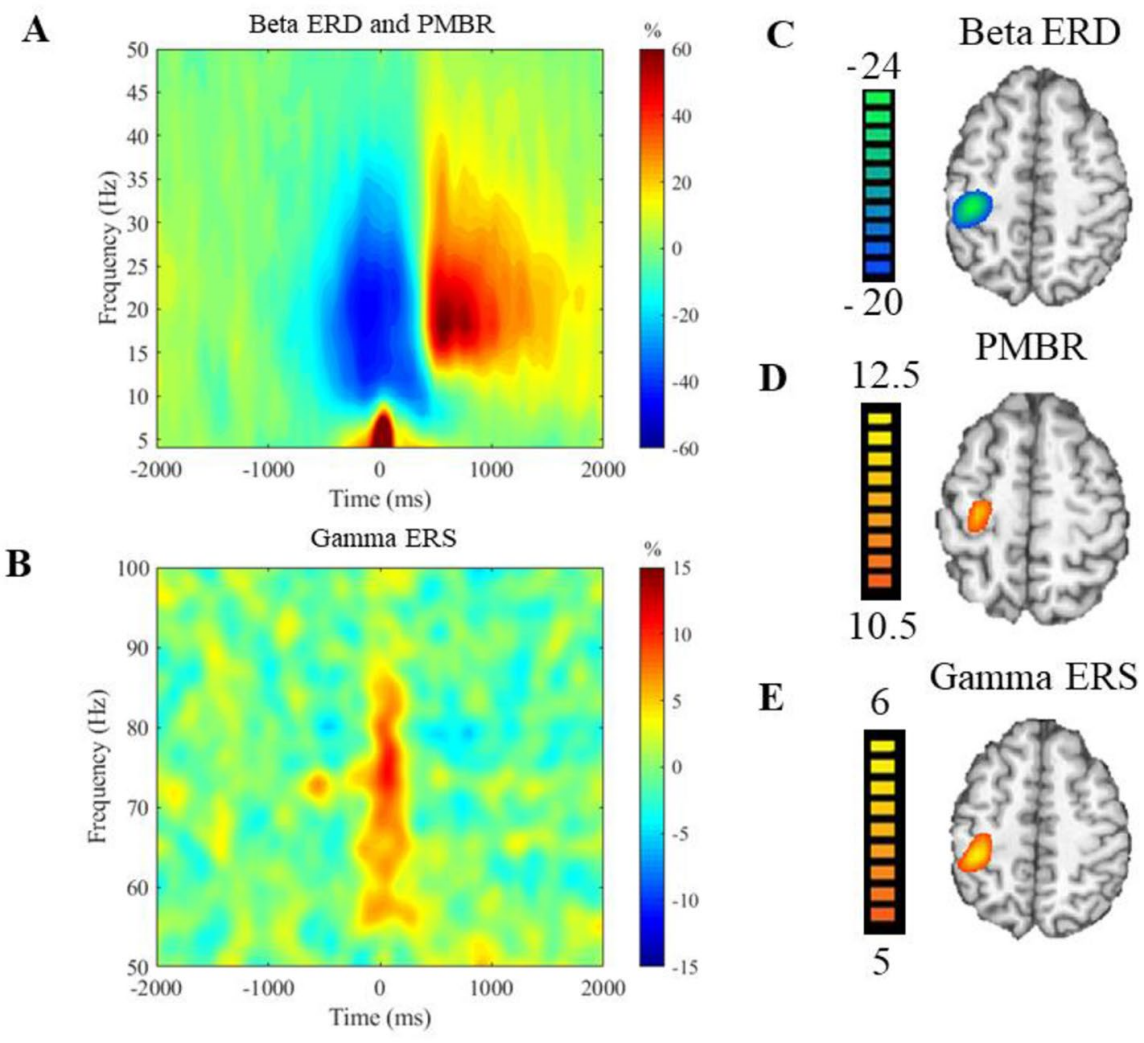
Sensorimotor spectrograms and group averaged beamformer images. (**A**) Averaged time–frequency spectrogram from a sensor located near the hand region of the contralateral (left) motor cortex averaged across all participants depicting the motor-related beta responses in the 0–50 Hz range. Time is denoted on the x-axis in milliseconds with 0.0 ms being movement onset, and frequency (Hz) is denoted on the y-axis. There was a beta ERD (18–26 Hz) that began about − 300 ms prior to movement onset and lasted until about 300 ms when the movement finished. After movement termination, there was a post-movement beta rebound (16–22 Hz) that lasted from about 500 to 800 ms before beginning to dissipate. (**B**) Averaged time–frequency spectrogram from a sensor located near the hand region of the contralateral (left) motor cortex depicting the 50–100 Hz range averaged across all participants. Beginning before movement onset around − 100 ms and lasting until about 100 ms after movement was a gamma ERS. Averaged beamformer images of the beta ERD (**C**) and the PMBR (**D**) depict that these responses were both located within the left sensorimotor cortices. (**E**) Group averaged image of the gamma ERS located in the contralateral (left) motor cortices.

For accuracy, there was a main effect of condition, consistent with the well-established “flanker effect,” which indicated that the accuracy was higher in the congruent condition in comparison to the incongruent condition (congruent = 97.2 ± 1.1%, incongruent = 96.2 ± 1.3%, P = 0.009; Fig. 2 and Supplementary Table 1). There was also a main effect of group indicating that the controls were more accurate than the adults with CP (CP = 93.5 ± 2.2%, NT = 99.4 ± 0.2%, P = 0.002; Fig. 3), and a main effect of sex (Males = 94.27 ± 2.47%, Females = 98.53 ± 0.50%, P = 0.019) indicating that females were more accurate than males. In addition, there was an interaction between group and condition (P = 0.010), with post hoc analyses revealing that the individuals with CP had reduced accuracy for the incongruent compared to the congruent condition (CP congruent = 94.64 ± 2.12%, CP incongruent = 92.43 ± 2.33%, P = 0.004). This was not the case for the controls (NT congruent = 99.44 ± 0.32%, NT incongruent = 99.44 ± 0.18%, P = 1.00). Finally, there was a significant interaction between sex and group, in which the males with CP had decreased accuracy in comparison to the females with CP (Males with CP = 88.67 ± 4.44%, Females with CP = 97.19 ± 0.85%, P = 0.019), but control males did not differ in accuracy from control females (NT Males = 99.07 ± 0.44%, NT Females = 99.72 ± 0.12%, P = 0.994). Regarding reaction times, there was a main effect of condition, again indicative of a flanker effect in which the reaction time was faster in the congruent compared to the incongruent condition (congruent = 597.4 ± 38.8 ms, incongruent = 653.0 ± 38.4 ms, P < 0.001; Supplementary Table 2). There was also a main effect of group indicating that the controls had faster reaction times than the adults with CP (CP = 754.4 ± 64.1 ms, NT = 512.2 ± 21.5 ms, P = 0.001). However, there was not a significant main effect of sex, and there were no interactions between the variables (P’s > 0.05).

### Sensor-level and beamforming analyses

Since only correct trials were used in the final analysis and the adults with CP had significantly fewer correct responses, trials were randomly removed from the controls to achieve similar signal-to-noise ratios between the groups. This was accomplished by randomly rejecting trials (i.e., sections of the raw data) in the control group to ensure that the total number of trials included in the final MEG analysis did not statistically differ between groups. The resulting number of trials per participant did not significantly differ between groups (CP = 172.43 ± 4.70, NT = 175.94 ± 1.65; P = 0.478).

Analysis of the sensor spectrograms collapsed across all participants and conditions revealed that there was a significant beta ERD (18–26 Hz; i.e., power decrease) across a range of sensors that covered the sensorimotor cortices, and that this response lasted from about -300 to 300 ms (P < 0.0001, corrected; Fig. 3A). There also was a significant PMBR (16–22 Hz; i.e., power increase) that occurred from 500 to 800 ms (P < 0.0001, corrected; Fig. 3A), and a significant gamma ERS (68–82 Hz) that occurred from − 100 to 100 ms (P < 0.0001; Fig. 3B). To identify the brain regions generating these responses, a beamformer was applied to each participant’s data using these time–frequency windows and a pre-stimulus window of equal duration and bandwidth.

### Beta ERD

**Figure 4:**
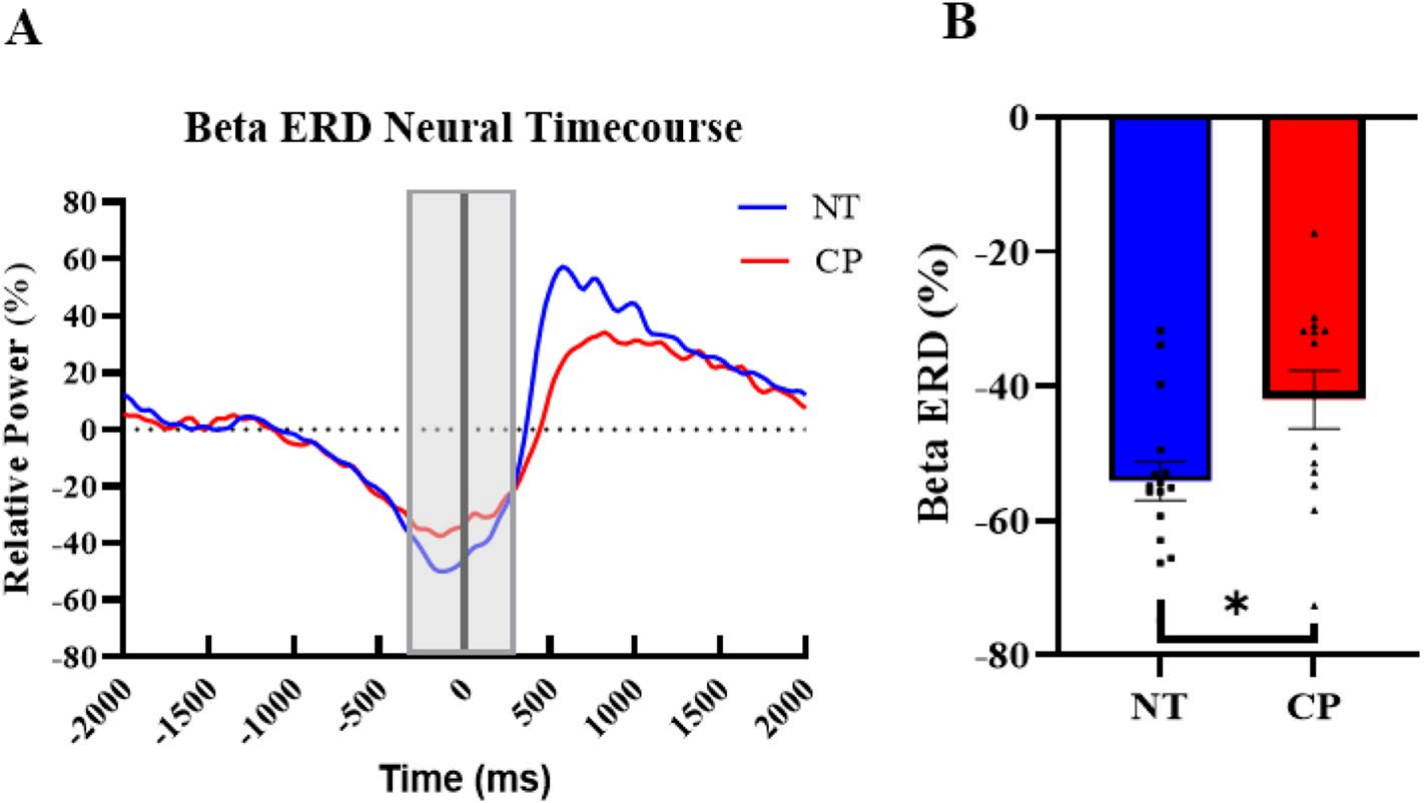
Beta ERD neural timecourse. (**A**) Neural timecourses of the beta ERD response in individuals with CP (red) and the NT group (blue) extracted from the peak voxel of the beta ERD response. The black line at 0.0 ms denotes movement onset, and the gray box is the window of interest (− 300 to 300 ms). (**B**) Bar graph depicting the difference in beta ERD strength between the NT and CP groups. As depicted, the beta ERD response was stronger in the NT group relative to those with CP. *P < 0.05.

The beta ERD (18–26 Hz) was imaged from − 300 to 300 ms with a baseline period of − 1600 to − 1000 ms. The grand averaged images showed that the beta ERD (Fig. 3C) was centered on the motor hand knob region of the contralateral (left) sensorimotor cortices. We subsequently extracted the timeseries from the peak voxel per participant and computed the magnitude of the response in the − 300 to 300 ms time window (Fig. 4A). Our statistical analysis revealed that there was a main effect of group, in which the adults with CP had a significantly weaker beta ERD compared to the NT controls (CP = − 42.0 ± 4.3%, NT = − 54.1 ± 2.9%, P = 0.026; Fig. 4B and Supplementary Table 3). However, there were no significant main effects of condition or sex, and there were no significant interactions (P’s > 0.05).

### PMBR

**Figure 5:**
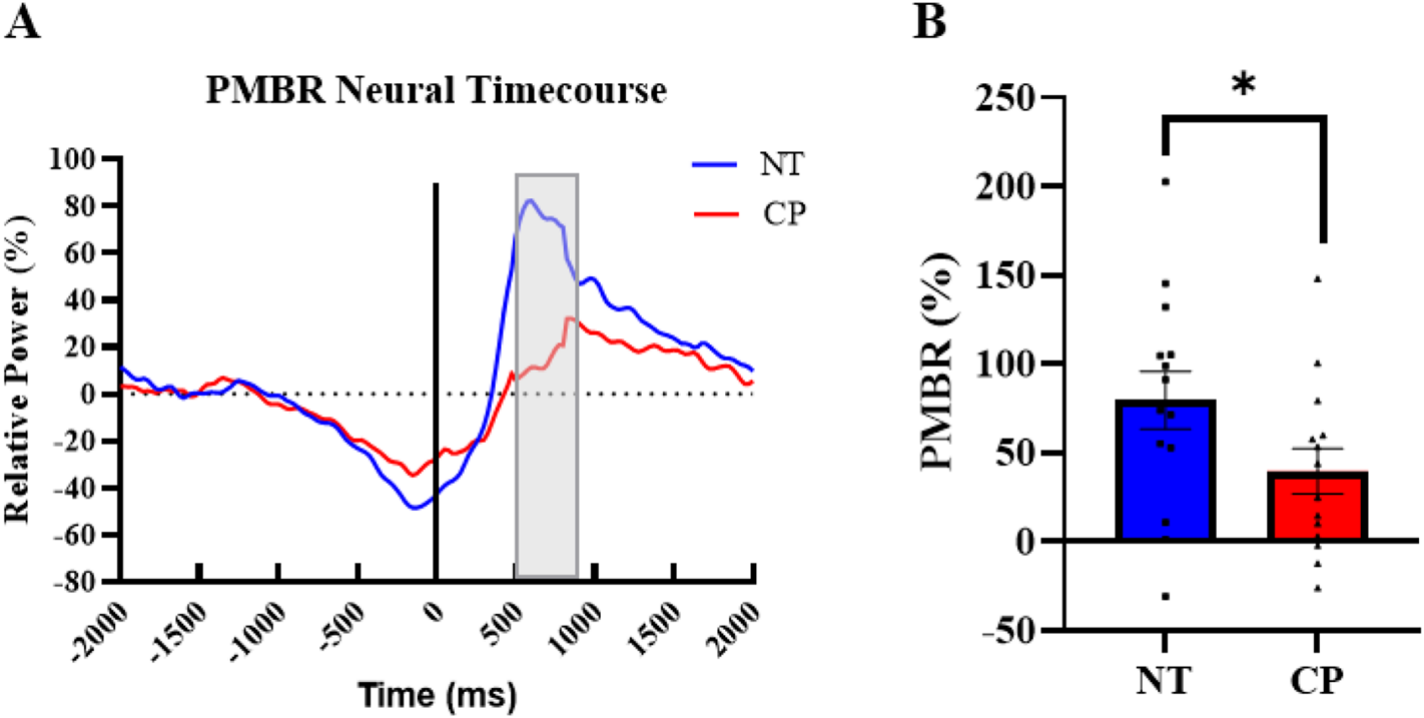
PMBR neural timecourse (**A**) Neural timecourses of the PMBR response in individuals with CP (red) and the NT group (blue) extracted from the peak voxel of the PMBR. The black line at 0.0 ms denotes movement onset, and the gray box is the window of interest (500 to 800 ms). (**B**) Bar graph depicting the difference in PMBR strength between the NT and CP groups. Overall, those with CP had a weaker PMBR compared to controls. *P < 0.05.

The PMBR (16–22 Hz) was imaged from 500 to 800 ms with a baseline of − 1600 to − 1300 ms. The grand averaged images showed that the PMBR was also centered on the motor hand knob of the contralateral (left) sensorimotor cortices (Fig. 3D). Next, we extracted the timeseries from the peak voxel of this image and computed the magnitude of the response in the 500 to 800 ms time window (Fig. 5A). There was a significant main effect of group indicating that the adults with CP had a significantly weaker PMBR in comparison to the healthy controls (CP = 39.6 ± 12.8%, NT = 79.6 ± 16.3% P = 0.043; Fig. 5B and Supplementary Table 4). There was also a significant main effect of sex, in which males had a significantly stronger PMBR than females (Males = 92.9 ± 16.7%, Females = 34.7 ± 11.1%, P = 0.004). However, there were no significant main effects of condition or significant interactions (P’s > 0.05).

### Gamma ERS

The gamma ERS (68–82 Hz) was imaged from − 100 to 100 ms with a baseline of − 1600 to − 1400 ms. The grand averaged images showed that the gamma ERS was also centered on the motor hand knob feature of the contralateral (left) motor cortex (Fig. 3E). Next, we extracted the timeseries from the peak voxel of this image and computed the magnitude of the response in the − 100 to 100 ms time window. Our statistical analyses revealed that there were no main effects of condition, sex, or group, and there were no significant interactions (P’s > 0.05; Supplementary Table 5).

### Spinal cord structure

**Figure 6:**
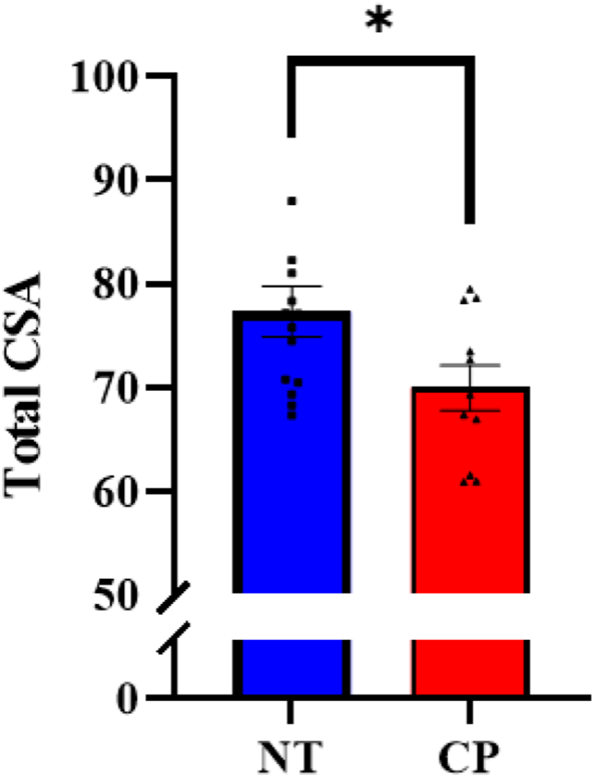
Total cross-sectional area (CSA) of the spinal cord. Individuals with cerebral palsy (CP) had a smaller total CSA than the neurotypical controls (NT). *P < 0.05.

Note that three individuals with CP and two controls did not complete spinal cord imaging. Thus, the sample size was 14 controls and 11 participants with CP for this aspect of the study. The total CSA of the spinal cord was smaller in the adults with CP compared with the controls (CP = 69.96 ± 2.15 mm^2^, NT = 77.30 ± 2.48 mm^2^, P = 0.020) (Fig. 6). In addition, the gray matter CSA (CP = 9.89 ± 0.81 mm^2^, NT = 12.43 ± 0.33 mm^2^, P = 0.002) and white matter CSA (CP = 63.06 ± 1.90 mm^2^, NT = 68.83 ± 2.43 mm^2^, P = 0.047) were also significantly lower in individuals with CP. When normalized to the total spinal cord CSA, the gray matter remained smaller in the adults with CP relative to the controls (CP = 15.0 ± 0.81%, NT = 16.9 ± 0.53%, P = 0.023), but the white matter no longer differed between the respective groups (CP = 90.7 ± 1.6%, NT = 88.9 ± 0.63%, P = 0.129).

### Correlation analysis

**Figure 7:**
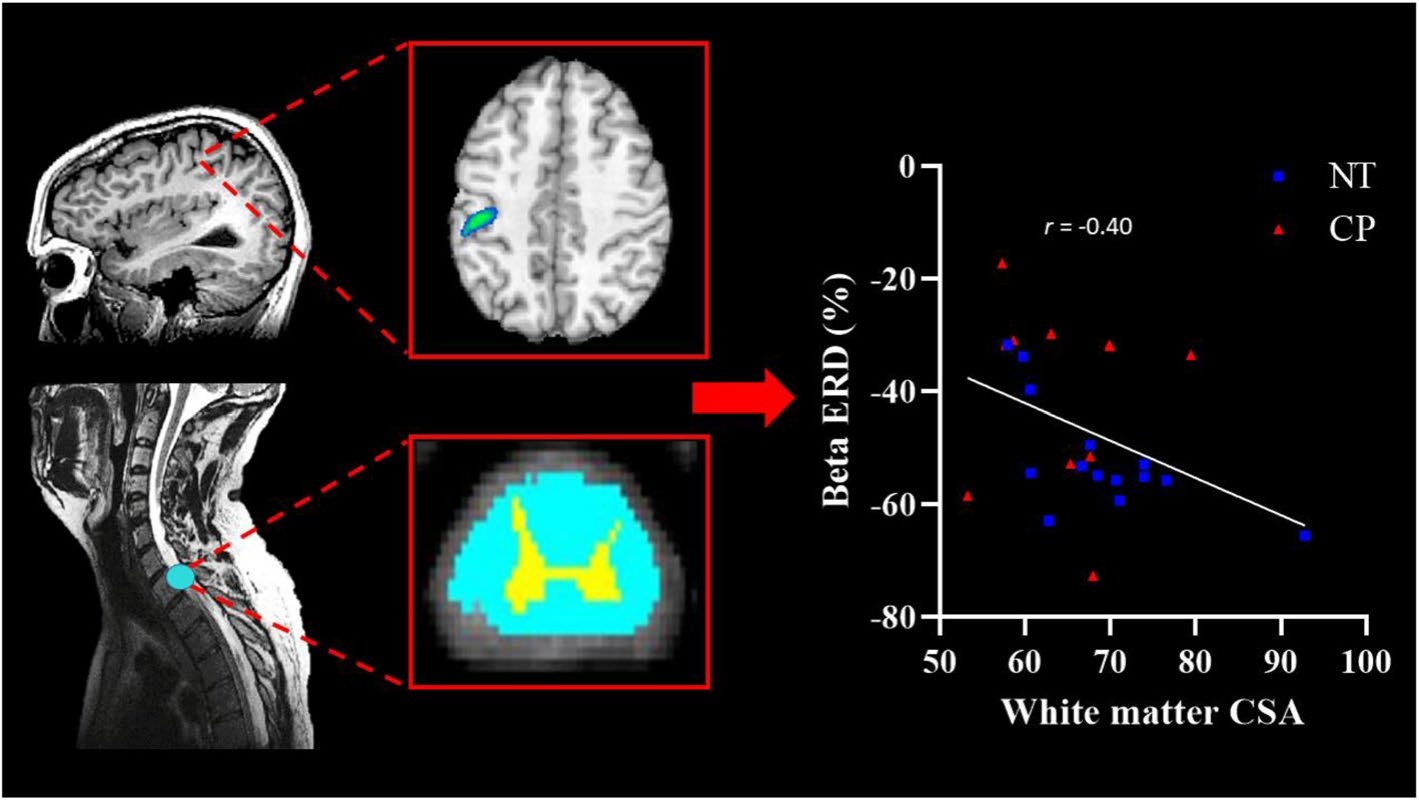
Cortical oscillations drive changes in spinal cord microstructure. *Left*: Representative T1 weighted images of the brain and spinal cord from an adult with CP. The light blue circles denote the area of the brain and spinal cord that the strength of the beta ERD and white matter cross sectional area (CSA) were taken, respectively (Talairach coordinates: − 42, − 24, 21; spinal cord section: C6–T3). *Middle*: Representative beta ERD response (top) and CSA of the spinal cord (white matter depicted in light blue and gray matter depicted in yellow). Right: Scatterplot depicting the relationship between the strength of the beta ERD and the white matter CSA. Individuals with a stronger beta ERD tended to have more white matter within their upper spinal cord (*r* = − 0.40, P = 0.048) and more total CSA (*r* = − 0.43, P = 0.031). Blue squares denote controls while red triangles reflect those with CP.

Reaction time was associated with the strength of the beta ERD (*r* = 0.40, P = 0.032) and the PMBR (*r* = − 0.39, P = 0.041), indicating that participants who had stronger beta responses tended to have quicker reaction times. The strength of the beta ERD was also associated with total CSA of the spinal cord (*r* = − 0.43, P = 0.031) and more specifically the white matter CSA (*r* = − 0.40, P = 0.048; Fig. 7), indicating that individuals who had greater myelination also tended to have a stronger beta ERD. No other correlations were significant (P’s > 0.05).

## Discussion

In the current investigation, we utilized a multimodal neuroimaging approach to evaluate the neurophysiological underpinnings of the aberrant cognitive-motor functions of adults with CP. Our results showed that the strength of both the beta ERD and the PMBR were reduced in the adults with CP compared to the controls. We also identified that the total CSA and white matter CSA of the spinal cord was partially linked with the strength of the beta ERD, which aligns with the premise that the spinal cord microstructure is affected by the strength of sensorimotor cortical oscillations. Further discussion of these novel findings is presented in the following sections.

One of our key findings was that the strength of sensorimotor beta oscillations was reduced in adults with CP relative to the controls. Furthermore, our results showed that a weaker beta ERD was associated with slower reaction times. These combined results indicate that the uncharacteristic motor actions seen in adults with CP may be partially attributable to the weaker sensorimotor beta oscillations. Interestingly, this finding differs from our prior studies in youth with CP, which have shown that the beta ERD is stronger in those with CP relative to their age-matched peers during lower extremity tasks^47–49^. This discrepancy may indicate that the neurophysiological mechanisms that govern cortical oscillatory activity during the respective motor tasks differ, such as differences in cognitive load and movement certainty, or it may be more reflective of developmental differences between youth and adults with CP.

Regarding mechanistic differences, the strength of beta oscillations is influenced by the number of interconnected glutamatergic pyramidal neurons that are collectively active. In this case, the stronger power reduction seen during the leg motor task might be related to fewer neurons oscillating at the beta frequency relative to the baseline. In other words, more neurons in the sensorimotor cortices switched to oscillating at a different frequency during motor planning and when execution of the leg motor action was initiated. Given that the nature of the insults seen in individuals with CP tends to cause greater disruptions in the leg region, the stronger beta ERD in this area might represent the necessity for a greater number of neurons to switch to the gamma frequency to execute the motor command. This premise seems to align with the TMS literature that has suggested youth with CP must generate a stronger cortical response to adequately excite the spinal motor neuron pools involved in the generation of leg muscular contraction^69^. Alternatively, we speculate that the weaker beta oscillations seen in this investigation for hand motor actions may be related to alterations in the γ-Aminobutyric acid (GABA) interneurons. Pharmaco-MEG studies with healthy controls have provided supporting evidence that an increased concentration of the inhibitory GABA neurotransmitter within the sensorimotor cortices results in a stronger motor related beta ERD^70,71^. Based on this scenario, the weaker beta ERD seen for the hand motor action of the adults with CP could also be related to reduced inhibitory interneuronal activity. While both alternative conjectures seem plausible, they need to be thoroughly tested to better understand the neurophysiological mechanisms at work during leg and hand motor actions. We propose that these potential alternative explanations could be tested through multimodal neuroimaging approaches that combine TMS, GABA spectroscopy, and the MEG methods employed in this investigation.

An alternative explanation is that the beta ERD differences seen for the leg and hand motor actions could be related to the cognitive load of the respective tasks. Prior research has shown that the beta ERD is stronger for movements with greater certainty^28,29,44^. In the prior lower extremity task mentioned above, the goal was to generate an isometric muscular contraction that matched a target force that was shown on the screen. While movement planning is still inherent, the fact that the movement is the same for each trial may create less of a cognitive challenge compared with the motor decisions that must be made for the Eriksen flanker task. In other words, the weaker beta ERD seen for the hand task might signify that the adults with CP had less certainty on the selected motor actions to be performed by the respective digits, while they have greater certainty in generating a simple leg motor action.

Finally, as noted above, a third possibility is that the discrepancy largely reflects a developmental effect. Essentially, prior research has shown that the strength of the beta ERD increases linearly with age in healthy controls^72^. Unfortunately, the developmental trajectory of the motor-related beta ERD response has not been established in individuals with CP. Potentially, the differences seen across the respective studies of youth and adults with CP might represent distinct trajectories of age-dependent changes in the sensorimotor cortical oscillations, and not the task differences per se. Future longitudinal studies are needed to discern the likelihood of this explanation.

Our results also revealed that the PMBR was weaker for the adults with CP. This result is well aligned with our prior work, which has shown that the PMBR is weaker in youth with CP compared with controls when performing the same Eriksen flanker task^50^. The PMBR is thought to reflect afferent sensory feedback upon completion of the motor action^31,32^ although other possible functions cannot be ruled out^33–37^. Thus, the adults with CP may have reduced feedback about the final outcomes of their motor actions. This reasoning aligns with the numerous investigations that have shown weaker somatosensory cortical activity in individuals with CP and connected this with their tactile acuity^73–82^. If sensory feedback is aberrant, then this may create serious difficulties in sensorimotor integration and decreased certainty in the feedforward predictions that are based on the internal model. Such a framework could partially explain the association between a weaker PMBR and the slower reaction times seen in this investigation. It has alternatively been suggested that a weaker PMBR may reflect inadequate inhibition of cortical networks once the motor action has been terminated^33–35^. Conceptually, this could also affect the fidelity of the motor actions seen in this patient population.

Our multimodal imaging illustrated that participants with a stronger beta ERD also tended to have more white matter CSA and total spinal cord CSA. Previous animal models illustrated that an insult to the developing brain leads to activity-dependent changes within the spinal cord^52,83,84^. Potentially, the weaker oscillatory activity seen in the sensorimotor cortices of the adults with CP noted in this study might contribute to maladaptive neuroplastic changes within the spinal cord that affect the myelination of the fiber tracts and synaptic connections. In other words, the integrity of motor cortical oscillations may have a downstream effect on the spinal cord microstructure. Ultimately, the fidelity of the hand motor actions in adults with CP might be dependent upon the coherent activity between the sensorimotor cortex and the spinal cord.

Somewhat surprisingly, we did not find any differences in the strength of the gamma ERS between the adults with CP and the controls. This went against our initial hypothesis, as our previous studies have repeatedly shown that the gamma ERS is reduced in youth with CP during both lower extremity tasks^47^ and the same Eriksen Flanker task^50^. Prior pharmaco-MEG studies have shown that a N-methyl-D-aspartate (NMDA) receptor antagonist can result in increased gamma ERS strength, while GABA agonists have no such effect^70,71,85^. Potentially, the amplitude differences seen across the respective studies for the youth and adults with CP might be NMDA receptor dependent. This would imply that the weaker gamma amplitude seen in the prior studies might be related to greater inhibition of the pyramidal cell population that play a role in the motor actions of youth with CP. Potentially, such NMDA receptor dysfunction may normalize with experience and age. Lastly, it is alternatively possible that the lack of group differences in this investigation may reflect the greater between-subject variation seen in prior studies of gamma oscillations^42^. As such, insufficient power may have played a role in our null effects.

In conclusion, we have expanded on previous literature by demonstrating that motor-related beta oscillations are weaker in adults with CP compared to healthy controls during tasks that involve cognitive-motor decisions. More importantly, we also found that the microstructural changes within the spinal cord of adults with CP appear to be related to the strength of sensorimotor cortical oscillations. These results suggest that altered sensorimotor cortical oscillations instigate activity-dependent plastic changes within the spinal cord. Ultimately, the alterations seen both in the cortex and the spinal cord likely interact to contribute to the upper extremity deficits seen in individuals with CP.

## Supplementary Information

Supplementary Information.

## Data Availability

The data that support the findings of this study are available from the corresponding author upon reasonable request.
