# Supplementary figures and images for: Spinal cord microstructural changes are connected with the aberrant sensorimotor cortical oscillatory activity in adults with cerebral palsy
Source: Sci Rep. 2022 Mar 21;12:4807. doi: 10.1038/s41598-022-08741-9 (PMC8938462; doi:10.1038/s41598-022-08741-9)

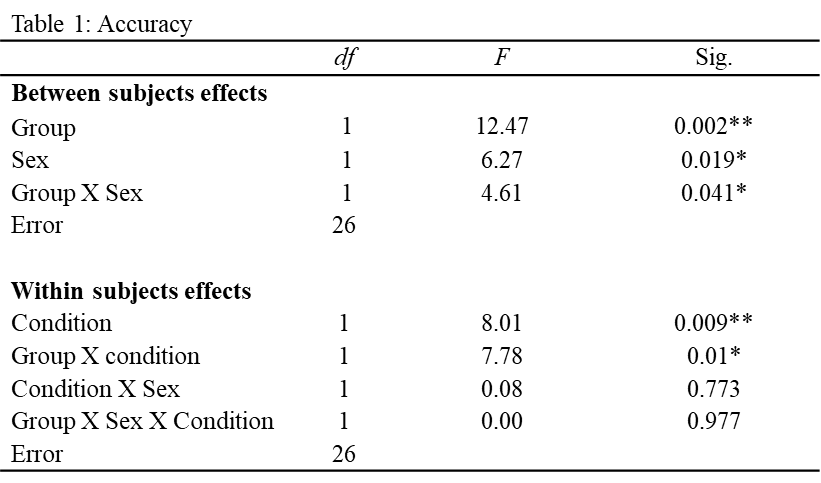
Supplemental material


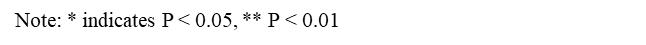


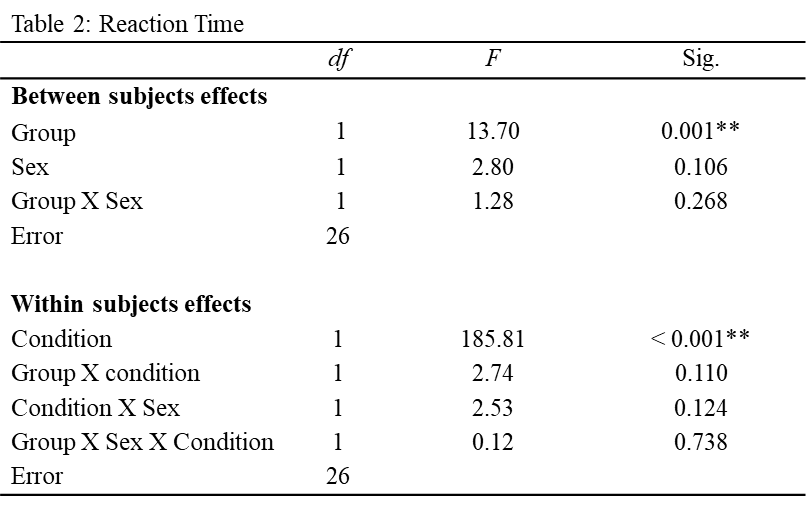


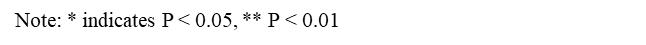


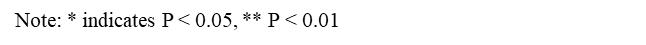


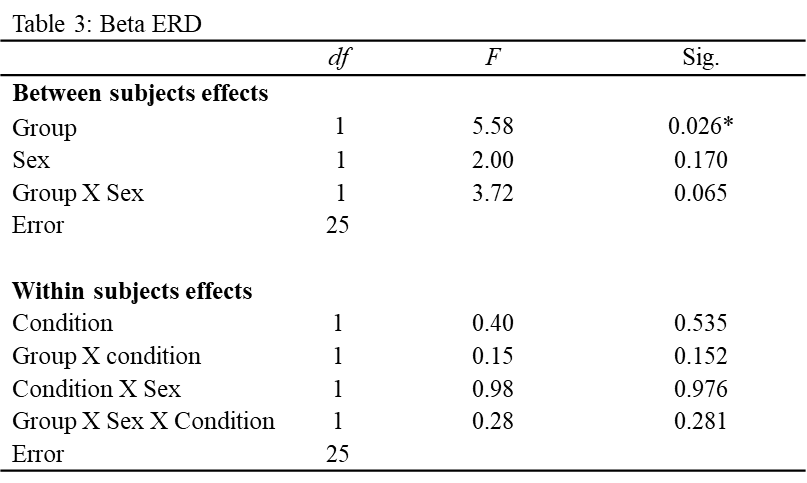


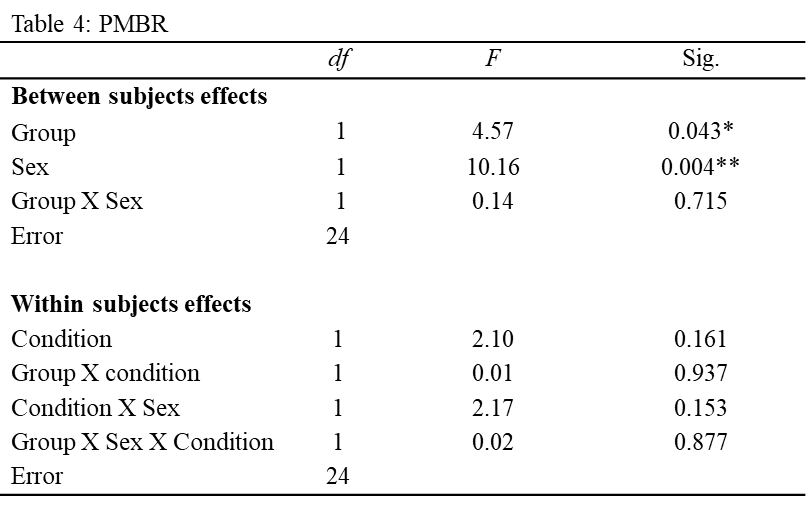


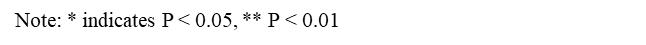


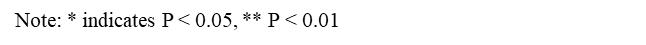


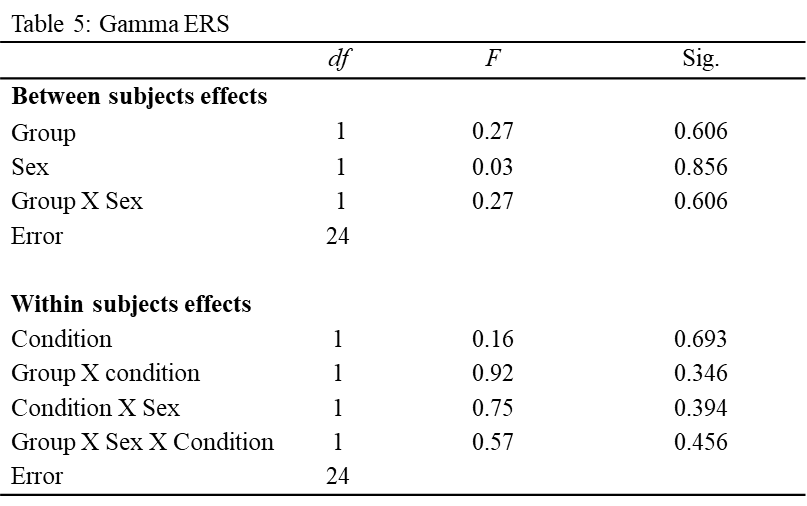

Supplement: Supplementary file 1 — Supplementary Information. [file 41598_2022_8741_MOESM1_ESM.docx]
